# Supplementary material for: Hydration of biologically relevant tetramethylammonium cation by neutron scattering and molecular dynamics
Source: Phys Chem Chem Phys. 2024 Jan 4;26(4):3208–18. doi: 10.1039/d3cp05449g (PMC10806622; doi:10.1039/d3cp05449g)
Supplement: CP-026-D3CP05449G-s001 [file CP-026-D3CP05449G-s001.pdf]

Electronic Supplementary Material (ESI) for Physical Chemistry Chemical Physics.
This journal is © the Owner Societies 2024

PCCP

ARTICLE TYPE

DOI:10.1039/c9cp05682c

ELECTRONIC SUPPLEMENTARY INFORMATION (ESI):

Hydration of biologically relevant tetramethylammonium cation by neutron scattering and molecular dynamics

Philip Mason*[a], Tomas Martinek[a], Balázs Fábián[a‡], Mario Vazdar[b], Pavel Jungwirth[a], Ondrej Tichacek[a], Elise Duboué-Dijon[c], and Hector Martinez-Seara*[a]

1 Additional details on neutron scattering

Measuring a total neutron scattering pattern for a solution yields an insight into the correlations between every atom in the system with every other atom in the system. While this is comprehensive, for most multi-atom systems it is too complex to interpret. Neutron scattering with isotopic substitution allows components of this to be isolated and examined separately. In the main body of the paper, we isolate the H_{TMA}-H_W component. However, other partial structure factors can be isolated from this experimental data. The most obvious to look at is the correlations between the H_{TMA} and all nuclei in the system that are not H_{TMA}. This is achieved by subtracting the H_{TMA}-H_W component from the first-order difference in the main body of the paper and is summarized in Eq. 1 in ESI. This is shown in real and reciprocal space in Fig. 1 in ESI. While a principal component of this function is the correlation between H_{TMA} and O_W on the solvating water, in practice it is very difficult to gain any useful insight from this function. This is because the intramolecular correlations between H_{TMA} and the other atoms on the TMA give very sharp peaks due to the molecular bonds. These very large peaks lie directly on top of the most interesting part of the solvation of the TMA ion in the 3–4 Å range.

$$\begin{aligned}\Delta S^{\mathrm{Y}}_{\mathrm{H_{non}}}(Q) &= 39.3 \cdot S_{\mathrm{H_{TMA}O}}(Q) + 6.5 \cdot S_{\mathrm{H_{TMA}C}}(Q) \\ &+ 2.3 \cdot S_{\mathrm{H_{TMA}N}}(Q) + 2.3 \cdot S_{\mathrm{H_{TMA}Cl}}(Q) \\ &+ 4.3 \cdot S_{\mathrm{H_{TMA}H_{TMA}}}(Q) - 54.6\end{aligned} \tag{1}$$

where Y refers to every atom in the system but exchangeable hydrogen atoms. This equation can be Fourier transform to the real space as:

[a] Institute of Organic Chemistry and Biochemistry of the Czech Academy of Sciences, Flemingovo nám. 542, 160 00 Praha 6, Email: philip.mason@uochb.cas.cz, hseara@gmail.com
[b] Department of Mathematics, Informatics, and Cybernetics, University of Chemistry and Technology Prague, Technická 5, 16628 Prague, Czech Republic
[c] Laboratoire de Biochimie Théorique, CNRS, UPR 9080, Institut de Biologie Physico-Chimique, 13 rue Pierre et Marie Curie, 75005 Paris, France
‡ Present address: Department of Theoretical Biophysics, Max Planck Institute of Biophysics , Max-von-Laue Straße 3, 60438 Frankfurt am Main, Germany

$$\Delta G^{\mathrm{Y}}_{\mathrm{H_{non}}}(r) = 39.3 \cdot g_{\mathrm{H_{TMA}O}}(r) + 6.5 \cdot g_{\mathrm{H_{TMA}C}}(r) + 2.3 \cdot g_{\mathrm{H_{TMA}N}}(r) + 2.3 \cdot g_{\mathrm{H_{TMA}Cl}}(r) + 4.3 \cdot g_{\mathrm{H_{TMA}H_{TMA}}}(r) - 54.6. \tag{2}$$

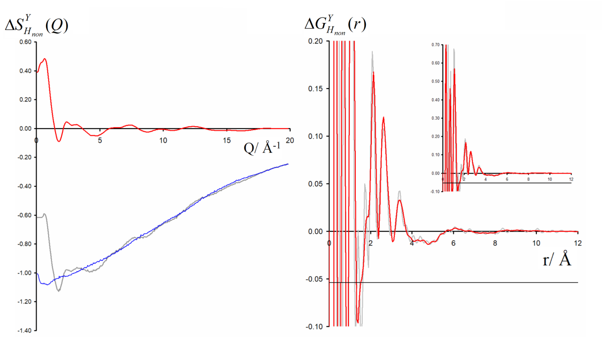

Fig. 1 Left in grey is the raw function $\Delta S^{\mathrm{Y}}_{\mathrm{H_{non}}}(Q)$. In the right, in grey is the direct Fourier transform of this function, $\Delta G^{\mathrm{Y}}_{\mathrm{H_{non}}}(r)$. The function $\Delta S^{\mathrm{Y}}_{\mathrm{H_{non}}}(Q)$ after background subtraction and smoothing is shown in red. In the right, in red is its direct Fourier transform $\Delta G^{\mathrm{Y}}_{\mathrm{H_{non}}}(r)$. As the H_{TMA}-C intramolecular peak of the TMA at 1.1 Å is not very interesting in $\Delta G^{\mathrm{Y}}_{\mathrm{H_{non}}}(r)$ and dwarfs everything else in this function, this is only shown in the inset.

2 Extra data AIMD

Contrary to all tested force fields, the AIMD simulation captures the shoulder in the experimental signal at low r. However, the box is too small to assess the signal quality at the position of the main peak and beyond. Still, the small size of the box may affect the RDF. The reasonable agreement with the experimental signal is obtained despite the AIMD simulation not containing any chloride counterion.

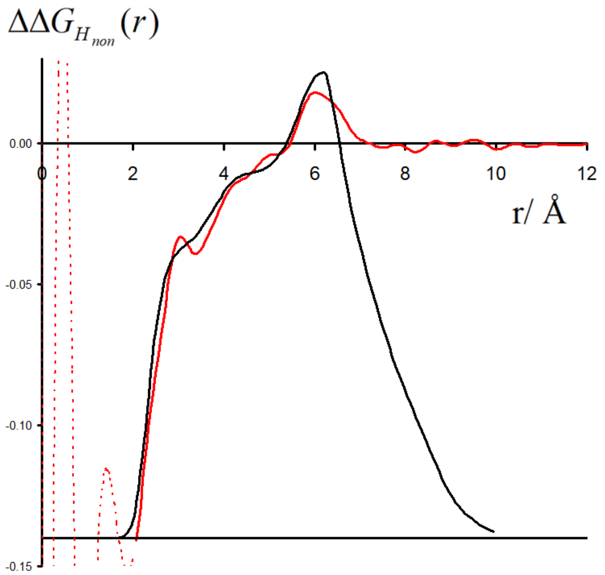

Fig. 2 Experimental Fourier-transformed signal $\Delta\Delta G_{\mathrm{H_{non}}}(r)$ (red), together with the same $\Delta\Delta G_{\mathrm{H_{non}}}(r)$ obtained from AIMD simulations (black).
